# The success-failure inversion in modern medicine

**DOI:** 10.3389/fpubh.2026.1932205

**Published:** 2026-09-07

**Authors:** Madhur Mangalam, Jessica Fabianiak, Barry Cense

**Affiliations:** 1 Department of Biomechanics, University of Nebraska at Omaha, Omaha, NE, United States; 2 Department of Ophthalmology and Vision Science, University of Nebraska Medical Center, Omaha, NE, United States; 3 Department of Electrical, Electronic and Computer Engineering, University of Western Australia, Perth, WA, Australia

**Keywords:** explanatory pluralism, geroscience, hallmarks of aging, healthspan, multimorbidity, philosophy of medicine, research funding, science policy

## Abstract

Twentieth-century medicine was most successful against diseases sharing a particular causal architecture: a discrete lesion standing in a relation of approximate necessity and sufficiency to a clinical syndrome, amenable to specific perturbation. Mortality from acute myocardial infarction has fallen by nearly 90 percent in the United States since 1970; pediatric leukemia has become survivable; HIV has become chronic. That success has shifted the residual burden toward conditions with a different architecture—late-life neurodegeneration, multimorbidity, frailty, idiopathic neuropathy—in which pathology is distributed across scales, and no single node carries the necessity that targeted intervention requires. The failure is not that medicine has extended life. It is that biomedical institutions have not adapted to the forms of vulnerability that accompany longer lives, and the non-adaptation is structural rather than accidental. This paper asks why: why funding and evaluation systems systematically favor research on discrete diseases and individual molecular targets over integrated research on aging as a systemic, multiscale, temporally extended process. We distinguish two levels at which the apparatus fragments its object. *First-order fragmentation* partitions aging into separately funded age-related diseases. *Second-order fragmentation* partitions aging itself into hallmarks, pathways, and targets pursued independently—which is why geroscience, the framework formulated to unify the age-related diseases, is decomposed by the apparatus that funds it. We specify the funding signatures each level of fragmentation predicts—across award duration, award mechanism, intervention modality, and the ratio of disease-specific to process-directed funding—and identify the data that would test them; we distinguish the several senses of reductionism at issue, defending integrative explanatory pluralism rather than holism; and we assess whether the hallmarks framework supports integration or licenses a longer list of targets. The argument is that the conceptual case for a multiscale account of late-life disease has been adequately made, and what remains unbuilt is institutional.

## A victory with unanticipated consequences

The cardiovascular successes of the 20th century were not merely therapeutic milestones; they were a reorganization of the human life course at the population scale. In 1968, age-adjusted mortality from coronary heart disease in the United States reached its historical peak and, against the predictions of most observers, began to fall (1). The decline continued for the next half-century. Between 1970 and 2022, age-adjusted mortality from acute myocardial infarction fell by approximately 89%, and from all ischemic heart disease by 81% (2, 3). Across the broader cardiovascular category, age-adjusted mortality fell by roughly 60% over the second half of the 20th century (4). The decline reflected coordinated advances spanning population-level risk factor reduction (tobacco control, dietary change, blood pressure management), pharmacological interventions (statins, antihypertensives, antithrombotics, and beta-blockers), and procedural innovations (coronary angiography, balloon angioplasty, stenting, coronary care units) (2, 3). The story is, in outline, a triumph of the localization framework: identify the lesion (atherosclerotic plaque, hypertension, dyslipidemia), specify the mechanism (lipid deposition, vascular injury, thrombosis), develop targeted interventions (lower lipids, lower blood pressure, stabilize plaques, reopen occluded vessels), and apply them at scale.

Parallel stories can be told for pediatric acute lymphoblastic leukemia, where 5-year survival rose from roughly 10% in the 1960s to over 90% through stepwise refinement of multi-agent chemotherapy (5, 6); for HIV, where combination antiretroviral therapy converted a uniformly fatal infection into a chronic condition with near-normal life expectancy (7, 8); for type 1 diabetes, where insulin replacement converted death within months into decades of managed life (9, 10); and for many cancers, where early detection and targeted therapy have shifted mortality curves substantially (11). Each victory shares a structural signature: few causal steps connecting a discrete lesion to a clinically defined syndrome, with the lesion amenable to specific perturbation. Where the signature holds, the localization framework delivers its characteristic results (12).

**Figure 1:**
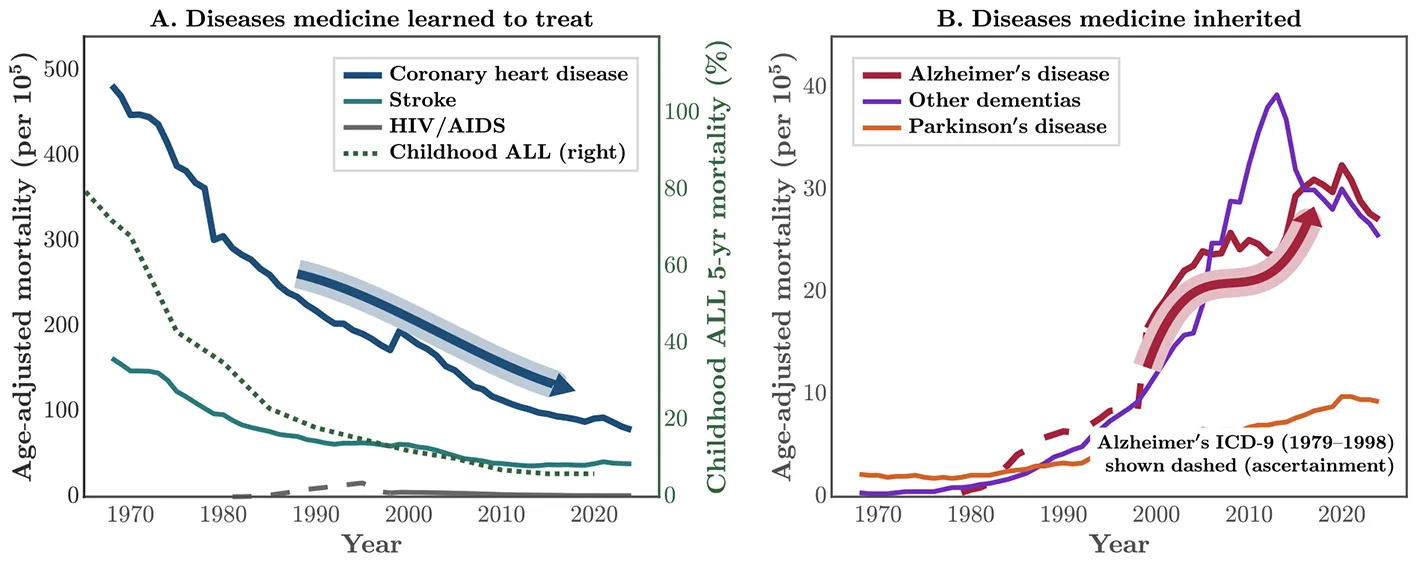
The success–failure inversion in cause-specific mortality, United States, 1968–2024. **(A)** Age-adjusted mortality from conditions tractable by localization declined substantially over the second half of the 20th century. Coronary heart disease mortality peaked in 1968 and fell by approximately 80% over the following five decades through coordinated advances in risk factor reduction, pharmacotherapy, and procedural intervention (2, 3). Stroke mortality declined by a comparable margin over the same period (4). Five-year mortality from pediatric acute lymphoblastic leukemia fell from above 80% in the early 1960s to below 10% by 2015 through stepwise refinement of multi-agent chemotherapy (5, 6); 5-year mortality is plotted on the secondary right axis. HIV/AIDS mortality rose sharply through the early 1990s and declined steeply after the 1996 introduction of combination antiretroviral therapy (7, 8). **(B)** Age-adjusted mortality from conditions whose pathology is not readily localized has risen in the surviving population over the same period. Alzheimer's disease and other dementias have moved into the leading causes of death in most high-income countries (15, 16), with Parkinson's disease mortality rising in parallel. The two trends are not independent: removal of early- and mid-life mortality from the conditions in panel **(A)** increases the population surviving into the age range at which the conditions in panel **(B)** present, and the conditions in panel (b) are precisely those for which the localization framework has not produced a transformative intervention. Data sources: CDC WONDER National Vital Statistics System, Underlying Cause of Death (Compressed Mortality 1968–2016, ICD-8/9; Bridged-Race 1999–2020, ICD-10; Single-Race 2018–2024, ICD-10); Children's Oncology Group trial data for pediatric ALL (5, 6); CDC MMWR and NCHHSTP surveillance reports for HIV/AIDS 1981–1998 (7). All mortality rates are age-adjusted to the 2000 United States standard population. The dashed segment for Alzheimer's disease (1979–1998) reflects ICD-9 code 331.0, introduced in 1979; the early rise in this segment partly reflects improved ascertainment on death certificates rather than a true change in incidence.

The aggregate effect is staggering. In 1900, average life expectancy at birth in the United States was approximately 47 years; today it exceeds 77 years, with most of the increase attributable not to slower aging but to the systematic prevention of deaths that previously occurred well before old age (13, 14). The framework has been so successful at picking the locks it can pick that, in much of the world, most people now reach an age at which they encounter the diseases whose locks it cannot (Figure 1).

The achievement is real, and the rhetorical structure of medical progress—“one disease conquered, another to follow”—suggests that the same approach will continue to deliver. We argue that this expectation overlooks a specific feature of disease architecture. The diseases medicine has solved are drawn from the subset whose architecture the localization framework was built to address. Solving them leaves the remaining diseases where they are. It shifts the population toward them by preventing the earlier deaths that would have occurred.

This has been argued before in various vocabularies, and we do not claim novelty. The question this paper takes as its object is the one that follows and has been addressed far less: given that the multiscale character of late-life disease is now widely acknowledged in the biomedical literature itself, why do funding and evaluation systems continue to select so consistently for research on discrete diseases and individual molecular targets over integrated research on aging as a systemic, multiscale, temporally extended process? The answer we develop is that the selection operates at two levels rather than one, that its second level captures even the frameworks formulated to escape the first, and that the mechanism is not intellectual conviction but the ordinary operation of evaluative criteria calibrated to a prior disease landscape.

The paper proceeds as follows. We first fix the causal distinction on which the argument depends and disambiguate the several senses of “reductionism” it involves. We then distinguish the two levels of fragmentation, specify the funding signatures each predicts and the evidence that would test them, and trace the mechanisms through which the apparatus reproduces itself. We turn next to what that apparatus produces at the point of care, to what an alternative apparatus would require in terms of both funding and diagnosis, and to whether the hallmarks framework escapes fragmentation or instantiates it at a second level. We close by distinguishing the descriptive claims our argument requires from the evaluative claims about aging and longevity that it does not.

## Where the deaths went

To see the inversion clearly, one must examine what survivors are now dying of. As deaths from cardiovascular disease, infectious disease, and many cancers have fallen, deaths from dementia and other neurodegenerative conditions have risen sharply in older populations, both absolutely and as a share of deaths: dementia has been the leading cause of death in the United Kingdom since 2015 and is now the leading cause among Australian women, and Alzheimer's disease has entered the 10 leading causes in the United States. Global estimates project a rise from roughly 57 million people living with dementia in 2019 to approximately 153 million by 2050, driven largely by population aging rather than age-specific incidence (15, 16).

Two interpretations of these numbers are common, and neither is adequate. The first treats the rise in dementia mortality as a coincidence of an aging population: people live longer, so more of them eventually develop dementia. The second treats it as a yet-unmet challenge that the next generation of pharmaceuticals will address, in the same way that statins addressed cardiovascular mortality and antiretrovirals addressed HIV. Both interpretations preserve the assumption that the localization framework is broadly correct and merely incomplete. Both miss what is structural about the situation.

The rise in dementia mortality has three separable components, and the argument requires careful attention to which is doing the work. The first is demographic: as the population over 75 grows in absolute and proportional terms, dementia deaths increase even if age-specific incidence is unchanged. The second is epidemiological: individuals who in earlier eras died of myocardial infarction at 62 now survive to 82, and late-onset Alzheimer's disease, whose median age of clinical onset exceeds 80, was not present in the truncated life course of the earlier cohort. The third is ascertainment: ICD coding for dementia improved substantially after 1979, and the early rise in Figure 1b partly reflects improved recording rather than true incidence change, as the figure caption notes. Each component contributes; the argument here rests primarily on the second. The probability of developing Alzheimer's disease conditional on surviving to age 85 is approximately 30%–35% (17); conditional on dying of myocardial infarction at 65, it is approximately zero. A program that converts the latter outcome into the former at a population scale mechanically increases dementia prevalence even if age-specific neurodegenerative biology remains unchanged. The framework shifted the population into the age range in which these diseases emerge—and these are precisely the diseases whose architecture it is least suited to address.

The historical record invites a second question: once the localization framework carries the population into diseases of advanced age, does its limited success reflect temporary scientific error or a structural mismatch between the framework and its new targets? Alzheimer's disease provides the clearest test case. For more than three decades, the amyloid hypothesis has organized research, drug development, and clinical trials around a localized pathological target, at a cost of tens of billions of dollars and with a clinical yield measured in weeks of delayed decline. Recent approvals of anti-amyloid therapies remain controversial because their benefits are modest and accompanied by substantial adverse events (18–20). Two interpretations follow. The first is contingent: amyloid was simply the wrong target. Under this view, a better-validated target would achieve for Alzheimer's disease what BCR–ABL inhibition achieved for chronic myeloid leukemia, and the history of failure reflects scientific misidentification rather than any limitation of the localization framework itself. The second is structural: Alzheimer's disease possesses no target whose manipulation is necessary and sufficient to reverse the syndrome. Successive candidates—amyloid, tau, neuroinflammation, the glymphatic system—therefore produce variations on the same outcome because each captures only one component of a distributed causal architecture. The first interpretation must explain why extraordinary investment repeatedly converged on inadequate targets over three decades. The second follows directly from the causal architecture developed in the previous section. A future intervention that substantially alters the course of Alzheimer's disease through a single localized target would falsify this second interpretation. Yet even such a result would leave the broader pattern unchanged: geriatric multimorbidity, frailty, idiopathic neuropathy, and chronic pain exhibit the same structural characteristics without a compelling candidate-target narrative to sustain the first interpretation. Similar histories appear elsewhere. The dopamine hypothesis of schizophrenia, the candidate-gene era in psychiatric genetics, and the monoamine hypothesis of depression each generated increasingly elaborate molecular accounts while clinical progress remained limited (21–23). Across these and related examples, the pattern is consistent (12): when disease architecture matches the localization framework, the framework succeeds; when it does not, the program expands its explanatory apparatus rather than its therapeutic reach.

Two clarifications keep the claim in its proper scope. First, the pattern has relatives—antimicrobial resistance, the hygiene hypothesis, the epidemiological transition (24–27)—but our claim is weaker than the familiar case of a superseded theory: the localization framework remains correct within its domain, and what its success changes is not its own validity but the distribution of problems that subsequently arrive. Second, that more people survive to ages at which neurodegeneration and multimorbidity present is a gain, and it is not the problem this paper identifies. Survival to advanced age is among the principal achievements of the past century, and nothing in the argument treats it as a cost to be regretted or an outcome to be traded away. The problem is that the institutions which produced that survival—funding mechanisms, trial methodology, regulatory categories, reimbursement structures, training pipelines—have not adapted to the forms of vulnerability that accompany it, and, as the remainder of this paper argues, the non-adaptation is structural rather than a matter of lag or insufficient attention. The failure is institutional and locatable, not demographic.

## Two architectures of disease

### What “reductionism” is doing in this argument

The term “reductionism” does at least six distinct jobs in discussions of this kind, and running them together produces an apparent opposition between reductionism and holism that we neither hold nor need. We therefore fix the senses at issue (Table 1) and state our commitments against them.

**Table 1:** Six senses of reductionism and the position taken here.

Sense	Claim	Position taken here
Ontological	Biological systems are constituted by, and nothing over and above, their physical parts	Accepted
Methodological	Decomposition into parts and their operations is a productive research strategy	Accepted; indispensable within an integrative program
Explanatory	Adequate explanation of a phenomenon is given at the lowest level of organization at which it can be stated	Rejected; explanatory adequacy is level-relative and phenomenon-relative
Causal localization	Pathology is attributable to a discrete node whose removal is necessary and approximately sufficient	A heuristic, valid for one class of disease architecture and not the other
Therapeutic	Effective intervention proceeds by identifying and perturbing that node	Conditionally valid; scope set by architecture, not by preference
Institutional	Specifiability of a single target functions as the criterion of fundability, approvability, publishability, and promotion	The object of this paper

We accept ontological reductionism without qualification, and we accept that methodological decomposition is indispensable—indispensable *within* an integrative program rather than merely alongside one, since multiscale characterization is not an alternative to decomposing systems into parts but a constraint on how the decomposed findings are recomposed. Our target is the inference from these two positions to a third: that because a process is materially molecular and methodologically decomposable, intervention at a single molecular node should be expected to produce clinically transformative effects. Causal localization is properly a heuristic, and the record of the 20th century establishes how productive a heuristic it is. This paper examines what happens when it hardens into an evaluative standard—when the specifiability of a single target becomes the criterion by which proposals are judged fundable, mechanisms are regulatory-grade, findings are publishable, and investigators are promotable. We designate this *institutional reductionism*. The positive position we defend is accordingly not holism but *integrative explanatory pluralism*: molecular mechanisms are retained and required, but no single level, target, or explanatory vocabulary is sufficient for the phenomena at issue, and recomposition across levels is itself a research object rather than a rhetorical gesture (28–32).

### Localized and distributed causal architectures

With those distinctions fixed, the pattern of success and failure traced above suggests a distinction between two classes of pathological architecture—a distinction in the causal-localization row of Table 1, not in the ontological one. The first class includes conditions in which a discrete molecular, cellular, or anatomical lesion stands in a relation of approximate necessity and sufficiency to a clinically defined syndrome. The BCR-ABL fusion gene drives chronic myeloid leukemia; phenylalanine hydroxylase deficiency produces phenylketonuria; HIV causes AIDS; LDL cholesterol deposition contributes causally to atherosclerotic plaque, whose disruption produces myocardial infarction. The second class includes conditions in which the pathological process is distributed across multiple scales and systems, with no single node having a necessary-and-sufficient relation to the syndrome that successful targeting requires. Late-onset Alzheimer's disease, Parkinson's disease, autism spectrum disorder, major depressive disorder, chronic pain, and most age-associated multimorbidity belong to this second class, and a substantial body of work now documents the multiscale character of their pathology (33–35).

The two architectures can be stated in terms that do not depend on treatment history, and the distinction therefore does not inherit the circularity of a classification based on which diseases medicine has and has not solved. A disease belongs to the first class if its clinical syndrome can be derived from a small number of causal steps, each carrying high necessity, connecting a discrete molecular or cellular event to the clinical presentation. The BCR-ABL fusion is necessary for chronic myeloid leukemia in a near-strict sense: the syndrome does not arise without the event, and the event reliably produces the syndrome in the relevant genetic background. HIV belongs to the same class: the retrovirus is necessary, its life cycle is defined, and removal of the retrovirus from the causal chain eliminates the disease. In the language of sufficient-cause models (36, 37), these conditions require few component causes, and the identifiable molecular event functions as a near-INUS condition in (38)'s sense—an insufficient but necessary part of a sufficient set that is itself close to minimal. A disease belongs to the second class if no such small sufficient-cause set exists: the relevant causal graph contains many nodes each carrying low individual necessity, with extensive feedback among them, such that removing any single node leaves the system capable of reaching the pathological state by alternative paths. Late-onset Alzheimer's disease satisfies this description: APOEε4 is the strongest known genetic risk factor yet is neither necessary nor sufficient—most carriers do not develop dementia, and many non-carriers do. The same holds for every other identified risk factor in isolation. The classification, therefore, rests on the structure of the causal graph, not on the history of attempts to intervene on it. That the classification correlates strongly with therapeutic success is consistent with the hypothesis that causal graph structure determines tractability; it is not a redescription of which diseases have happened to be solved. We acknowledge that the classification carries residual ambiguity when the causal graph structure is itself uncertain—which holds for most conditions in the second class, since the full graph is unknown. The claim is not that every disease can be assigned to a class with precision, but that the distinction is principled and prior to treatment history, and that the observed pattern of success and failure is what the distinction predicts.

**Figure 2:**
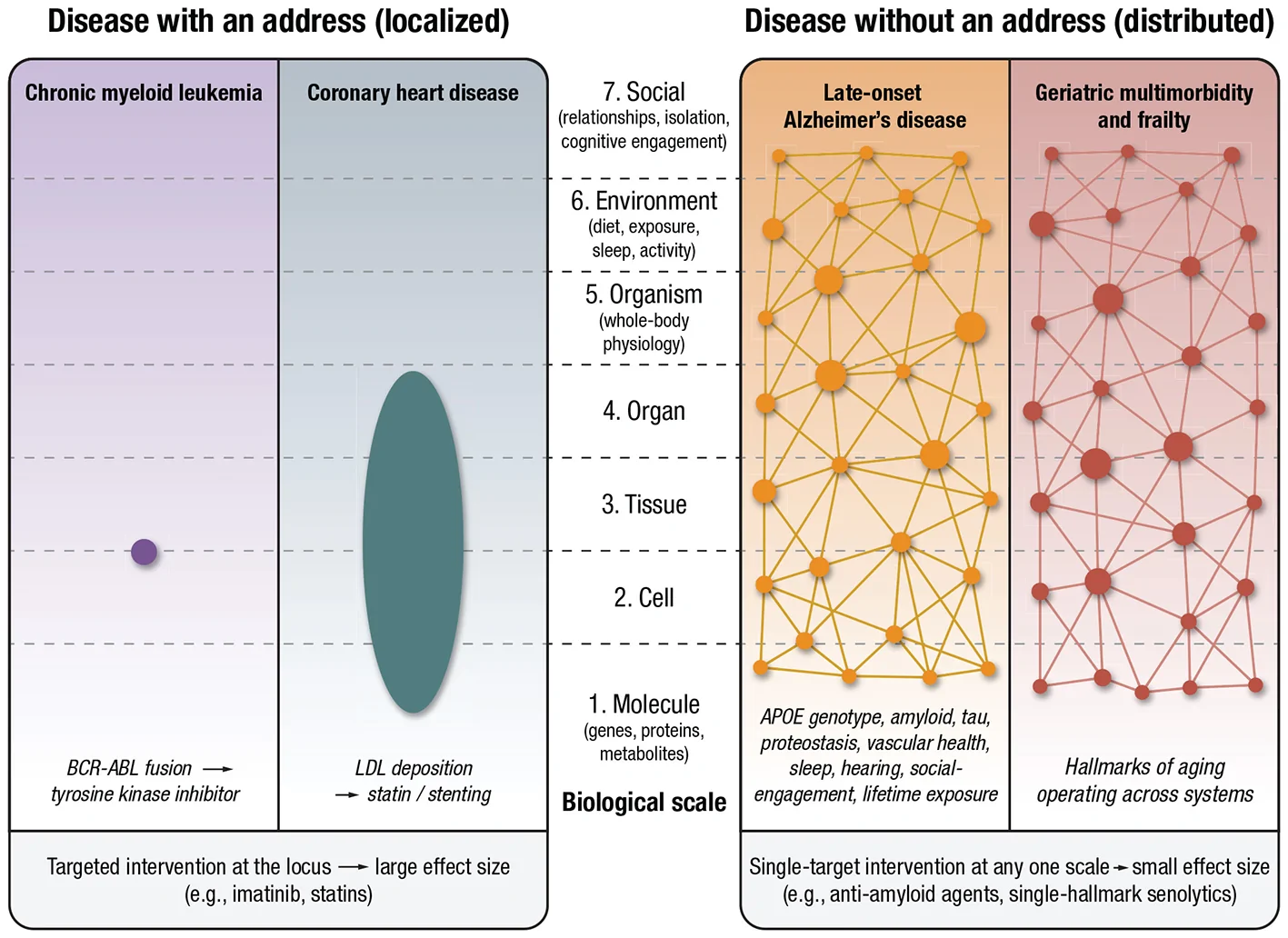
Two architectures of disease. Conditions tractable by the localization framework of 20th-century medicine **(left)** share a common structural signature. Their pathology localizes to a relatively narrow band of biological scales, allowing targeted intervention at that band to produce large clinical effects. Chronic myeloid leukemia represents the limiting case of this architecture: a single molecular event—the BCR-ABL fusion—produces a cellular phenotype that is necessary and approximately sufficient for the syndrome, and targeted kinase inhibition correspondingly restores function in many patients. Coronary heart disease spans from the molecular level through organs, including lipid deposition, endothelial dysfunction, atherosclerotic plaque formation, and arterial occlusion. However, its pathology remains largely confined to the lower tiers of the biological-scale axis, and interventions at those tiers, such as statins and stenting, yield major reductions in mortality. Conditions that resist localization **(right)** exhibit a fundamentally different architecture in which pathology is distributed across scales ranging from the molecular to the social. In these conditions, no single node or scale carries the necessary and sufficient relation to the syndrome that targeted intervention requires. Late-onset Alzheimer's disease involves APOE genotype, amyloid and tau pathology, proteostasis loss, vascular contributions, sleep architecture, sensory function, social engagement, and lifetime exposure operating across decades. Geriatric multimorbidity and frailty similarly involve the hallmarks of aging acting simultaneously across multiple systems, with extensive feedback among components, such that single-target interventions at any one scale tend to produce only modest effects, and the institutional apparatus designed to generate such interventions is structurally ill-suited to producing the multimodal alternatives the architecture would require. Only the vertical axis (biological scale) carries interpretive meaning; horizontal position within each panel is non-quantitative and serves to display multiple components within the available space. The network representation in the right-hand panels is illustrative rather than exhaustive; the relevant components and interactions are unlikely to be fully identified. Likewise, the drug examples shown in the intervention boxes are representative rather than comprehensive.

The boundary is fuzzy, and many conditions sit at intermediate positions. Type 2 diabetes is instructive: insulin resistance is central and pharmacologically tractable, yet the condition involves coordinated dysregulation across beta-cell function, hepatic glucose handling, adipose inflammation, the gut microbiome, lifetime metabolic exposure, and sleep architecture (39). Class membership matters less than the operational asymmetry: when a disease localizes, targeted interventions yield large effects; when it does not, they yield small ones, and the field's response has historically been to refine the molecular hypothesis rather than reconsider the architecture. That the first class is precisely what medicine has succeeded against, and the second precisely what it has not, is not a tautology; it reflects a structural fit between program and target (Figure 2).

The geroscience framework proposes that the major chronic diseases of late life share underlying biology in the form of the hallmarks of aging: genomic instability, telomere attrition, epigenetic alteration, loss of proteostasis, disabled macroautophagy, deregulated nutrient sensing, mitochondrial dysfunction, cellular senescence, stem cell exhaustion, altered intercellular communication, chronic inflammation, and dysbiosis (40, 41). Because the framework is both the most developed available response to the architecture described here and, we will argue, the clearest case of what the funding apparatus does to such responses, we defer its assessment to a dedicated section below.

Table 2 summarizes the success–failure inversion. The pattern is consistent enough that it should be regarded not as a contingent feature of medical history but as a structural prediction of what the localization framework would and would not deliver if pursued with sufficient resources and time. We now have those resources and that time, and the question that follows is institutional.

**Table 2:** The structural sorting of disease by localizability.

Condition	Architecture	Tractable?	Status
Coronary heart disease	Localized plaque, modifiable risk factors	Yes	~80% mortality decline 1970–2022
Acute lymphoblastic leukemia (pediatric)	Defined molecular subtypes	Yes	Five-year survival >90%
HIV/AIDS	Single retrovirus, defined life cycle	Yes	Near-normal life expectancy on cART
Type 1 diabetes	Autoimmune beta-cell loss	Partial	Replacement therapy enables decades of life
Many bacterial infections	Single organism, defined pathway	Yes	Antibiotics; prognosis transformed
Late-onset Alzheimer's	Multiscale, polygenic, lifetime exposure	No	Marginal benefit despite >30 years of effort
Parkinson's disease	Multisystem, decades-long prodrome	No	Symptomatic dopamine replacement only
Major depressive disorder	Heterogeneous, network-like	No	Modest effects above placebo, no consistent biomarker
Frailty/sarcopenia/multimorbidity	Multisystem decline across hallmarks of aging	No	No single intervention; multidomain approaches emerging
Chronic pain/fibromyalgia	Nociplastic, multiscale	No	Multimodal care; pharmacological yield poor

For each condition, the table records the dominant pathological architecture, whether the localization framework has produced a transformative intervention, and the typical clinical outcome. The conditions in the upper rows have been substantially addressed by 20th-century medicine. The conditions in the lower rows remain largely unaddressed, with the bulk of contemporary mortality and disability burden shifting toward them as the upper conditions have been controlled.

Two objections follow naturally and are worth answering before the institutional argument proceeds. The first is counterfactual: would medicine have done better had it pursued a systemic program from the outset? Our answer is no, and the claim of this paper does not require otherwise. For conditions with the architecture described in the left column of Figure 2, localization was not merely one available strategy, but the correct one, and the effect sizes it delivered—an eighty percent reduction in coronary mortality, a chronic disease made of a fatal infection—are not obtainable by a program that declined to identify discrete targets where discrete targets exist. A systems-first 20th century would have been a worse one. Nothing here argues for replacing targeted intervention; the argument concerns what is required in addition when the architecture differs.

The second objection is sharper: does the distinction we draw concern the causal structure of diseases, or merely the methods used to study them? It concerns the former, which is why we have defined the two classes in terms of causal-graph properties specifiable prior to and independently of any treatment history. A monogenic driver and a set of many low-necessity interacting contributors are fundamentally different causal objects, not different descriptions of one object. The method that succeeds against the first is not inadequate to the second so much as inapplicable: there is no node whose removal would do what removing BCR-ABL does. The methodological consequence follows from the causal fact rather than substituting for it.

We should also be explicit about what the alternative has so far demonstrated, since it would be inconsistent to fault the dominant program for overclaiming and then overclaim on behalf of its rival. The best-evidenced multidomain results are modest (42–44), and the effects of resistance training and protein adequacy on function in older adults, while substantial (45–47), are not of the magnitude that statins achieved in coronary disease. We do not claim that a multiscale program will match the effect sizes of 20th-century localization. We claim that, for the residual conditions, no single-target approach has produced better results despite far larger investment; that the combinatorial hypothesis has not been tested under designs capable of adjudicating it; and that the absence of those tests is explained by the institutional analysis that follows. A demand for demonstrated effect sizes before a program is funded is, in this context, an instance of the selection mechanism rather than a neutral standard applied to it.

## First- and second-order fragmentation

The institutional argument requires a distinction that the disease-architecture account does not by itself supply. The apparatus fragments its object at two levels, and the second determines whether an integrative alternative can survive contact with the funding system.

*First-order fragmentation* partitions aging into age-related diseases that are studied, funded, trialed, and reimbursed separately: Alzheimer's disease, coronary artery disease, osteoporosis, sarcopenia, and several cancers, which are constituted as distinct objects with distinct institutes, appropriations, professional societies, endpoints, and regulatory pathways. This is the fragmentation that the geroscience framework was explicitly formulated to overcome (40, 41), on the grounds that the conditions share upstream biology and that intervening in that biology would address them jointly rather than serially.

*Second-order fragmentation* partitions aging itself. Once the shared upstream biology is specified as a list of hallmarks, pathways, or processes, the apparatus applies to that list the same evaluative criteria it applied to the disease list: a proposal is legible when it names one hallmark, specifies a mechanism of action within it, and proposes an intervention adjudicable against a pre-specified endpoint within a funding cycle. Senolytics address cellular senescence; NAD^+^ precursors address deregulated nutrient sensing and mitochondrial function; partial reprogramming addresses epigenetic alteration. Each is a single-target program with a single mechanism of action, and each is therefore fundable, patentable, trialable, and promotable in a way that simultaneous perturbation across several hallmarks is not.

The point we wish to press is that these are one mechanism operating at two levels. The same criterion—specifiability of a discrete target within a bounded time horizon—produces both. This is why a framework can be, as geroscience is, an explicit and well-motivated response to first-order fragmentation while undergoing second-order fragmentation almost immediately upon acquiring institutional traction, and why that decomposition follows directly from the incentives themselves. The investigators need only have written fundable proposals. The three transitions can accordingly be stated as a sequence, each driven by the same evaluative pressure: from aging to individual age-related diseases; from those diseases to isolated mechanisms within them; and, once an integrative framework displaces the first transition, from that framework to interventions directed at one of its elements. The apparatus assimilates integrative frameworks and returns them in a decomposed form. Table 3 states the two levels and the funding signature each predicts.

**Table 3:** Two levels of fragmentation and their predicted funding signatures.

Level	What is partitioned	Rationale offered	Predicted funding signature
First-order	Aging → discrete age-related diseases	Disease-specific expertise, endpoints, constituencies	Disease-designated appropriations dominate process-directed funding; burden–effort mismatch persists
Second-order	Aging → hallmarks, pathways, single targets	Mechanistic tractability, regulatory legibility, patentability	Single-hallmark awards dominate combinatorial ones; factorial designs near-absent; behavioral and multidomain trials underfunded relative to pharmacological ones

Each level generates a testable prediction about the distribution of research funding; we specify below what data would evaluate them.

We state plainly that the present paper specifies this test rather than performing it. The predictions in Table 3 are empirical and could be evaluated against project-level records in NIH RePORTER, NIA budget mechanism tables, CORDIS, UKRI Gateway to Research, and ClinicalTrials.gov sponsor metadata, benchmarked against disease burden using the framework of (48). One obstacle is worth naming because it is itself part of what we describe: no major funding taxonomy contains a category corresponding to aging as an integrated process, so any quantification requires an explicit operationalization whose coding decisions others might reasonably make differently. We regard the construction of that operationalization and the analysis it would enable as the natural next step for this argument rather than as a step this paper takes.

## How the apparatus reproduces the fragmentation

Neither level of fragmentation is sustained primarily by intellectual conviction. What sustains both is an institutional architecture whose evaluative criteria select, at every stage, for proposals that name a discrete target within a bounded horizon. The criteria are neither arbitrary nor malicious; each was calibrated to a disease landscape in which they were appropriate, and each remains reasonable in isolation. Their conjunction is what produces the effect described here.

A natural objection is that the mechanisms can be adapted, and to a degree, they have been. Multi-investigator awards, longitudinal cohorts, consortium programs, and large-scale data integration all exist; NIH multimorbidity initiatives, the NIA-led geroscience programs (40), and international consortia on common mechanisms of aging have funded work explicitly framed across systems and conditions. The objection has force, and the contributions are real. The adaptations have nonetheless remained a small fraction of total biomedical funding, and—the more telling point—their evaluation criteria still, in practice, favor projects that can articulate a specific mechanism, even when the framing invites integration. The accommodations are made within an evaluative regime that they do not alter. Four features of that regime deserve separate statement.

### Specifiability

The dominant funding mechanism—the NIH R01 and its structural analogs elsewhere—is organized around a discrete hypothesis, a defined set of experiments, and outcomes adjudicable within 3–5 years by a single principal investigator. The specificity is a feature: it disciplines investigators to articulate testable claims and permits reviewers to assess feasibility. But it becomes an obstacle when the phenomenon is multiscale and its time course spans decades. A proposal to characterize the molecular pathology of amyloid clearance can state in advance what will be measured; a proposal to characterize how microbiome composition, sleep architecture, social engagement, vascular health, and cognitive activity jointly shape neurodegenerative trajectories cannot, and the investigators are not at fault: the phenomenon does not admit it. The predictable response is to ask that the proposal be narrowed, and investigators who would like to be funded comply. The compliance determines what is measured, what is published, and what the next cohort of reviewers is trained to recognize as rigorous and fundable (49, 50). The grant written to be fundable produces the literature that validates the criteria by which future grants are funded.

### Novelty

Reviewers are asked to assess innovation, and the criterion operates asymmetrically across the two disease architectures. For localizable conditions, a new molecular target is genuinely new information that extends the capacity to intervene. For multiscale conditions, the most accurate framings are decades old—the biopsychosocial model dates from 1977, the hallmarks framework from 2013—and cannot be rendered novel without becoming less accurate. The investigator committed to an accurate framing must present it as a methodological or computational advance rather than a conceptual one; the investigator proposing a new target within the established paradigm claims genuine novelty at the cost of repeating a claim structure that has repeatedly failed. The criterion thus rewards the less accurate framing in precisely the disease class where accuracy matters most: the better a framing has held up, the harder it is to fund work within it.

### Time

Processes that take decades to develop require study measured in decades. The apparatus operates in cycles of 3–5 years, so longitudinal infrastructure must be sustained across multiple cycles, often multiple agencies, under criteria that shift between them. The cohorts that have managed it—Framingham, Whitehall, Rotterdam, Honolulu-Asia—were built laboriously and have survived funding crises through institutional commitment and individual persistence rather than through any mechanism designed to sustain them. Comparable infrastructure for the multiscale architecture of late-life disease is not being built at a pace commensurate with the burden.

### Modality and career

The journal and regulatory apparatus reinforce the same selection from different directions. High-prestige venues reward work that identifies a mechanism, demonstrates a functional consequence, and reverses it by targeted intervention in a model system; regulators require specifiable mechanisms of action, defined populations, and pre-specified endpoints. The cumulative effect is to exclude work whose mechanisms are distributed, whose populations are heterogeneous, and whose effects develop over time horizons longer than those of a standard trial. The FINGER family of trials, which produced the most credible evidence that multidomain intervention can slow cognitive decline, had to develop trial methodology from first principles, are unpatentable in the sense the regulatory system prefers, and have attracted sustained funding only with difficulty (42–44). Career structure enforces the same logic on an individual level. Academic advancement is built on first-authored papers, single-investigator awards, and a scientific identity attached to a defined topic; the investigator studying how the microbiome, sleep, isolation, and inflammatory tone jointly shape cognitive trajectories faces, at every review, the question of what, specifically, she is an expert in. The acceptable answers map onto localization. The training pipeline produces the investigators that the funding apparatus can evaluate, and the funding apparatus rewards the projects that the pipeline prepares them to conduct.

These four features stabilize one another, which is why marginal adjustments in any one of them are absorbed by the others. Funding levels for individual disease areas continue to correlate poorly with disease burden across multiple metrics, and the mismatch has persisted for two decades despite repeated recommendations for realignment (51, 52). Nor is it a peculiarity of the United States appropriations process: (48) document the divergence between the global distribution of research effort and the global distribution of disease burden, indicating that the selection described here operates on research systems generally rather than on a single national architecture.

## Why the apparatus does not register the anomalies

The self-stabilization just described has an epistemic counterpart, and Lakatos's account of degenerating research programs names it precisely (53). A research program on this account consists of a hard core protected by a belt of auxiliary hypotheses; it is progressive when modifications anticipate new facts, degenerating when they accommodate anomalies afterward without extending predictive reach. The localization framework was progressive in this sense for much of a century. The record of the past three decades in neurodegeneration, psychiatric genetics, and chronic pain suggests a program that has crossed into the second condition: the amyloid cascade hypothesis has absorbed failed trials, revised targets, and successive stratification schemes through modifications accommodating results without altering the program's predictions (18–20), and the monoamine hypothesis of depression and the candidate-gene framework in psychiatric genetics exhibit the same structure (21–23).

The second half of Lakatos's account matters more here. A degenerating program is rarely overturned by decisive experiment, because its protective belt is flexible enough to absorb almost any result; what displaces it is a rival program with greater progressive potential. The localization framework has no such rival in place. The biopsychosocial model (35), network theories of psychopathology (33), the Research Domain Criteria framework (54), and geroscience (40, 41) each represent partial attempts at an alternative architecture, and none has matched the incumbent's conceptual clarity, methodological tractability, regulatory acceptability, and clinical actionability. Widespread acknowledgment of multiscale architecture, therefore, coexists with a research practice still organized around localization—not because investigators are unaware of the mismatch, but because the degenerating program remains, institutionally, the only one within which a career can be built.

This is what makes the situation unusual. The anomalies are conspicuous and extensively documented; the argument for the multiscale architecture of late-life disease has been adequately made at the level of ideas. The open question is not whether the alternative framing is correct but whether a rival program can be built that matches the incumbent's institutional purchase, which is an organizational and political-economic question as much as a scientific one.

What the apparatus produces is legible at the point of care. Box 1 describes a composite patient who is both the product of the 20th century's successes and the defining test case for the 21st century's difficulties. The institutional architecture that produced her was built to localize, isolate, and treat discrete pathologies; her condition instead spans interacting physiological, behavioral, and environmental processes across multiple timescales. Recognizing that mismatch does not produce the clinics, reimbursement codes, training programs, trial designs, or regulatory pathways required to address it. Those institutions must be designed for distributed disease architectures rather than localized lesions, and the next advance requires an institutional framework organized around a different conception of disease.

Box 1 **The arithmetic of the new patient**

A woman arrives at the geriatrician's office in 2026, aged 84, having survived the diseases the 20th century learned to treat. A statin has contributed to her not dying of myocardial infarction at 65; an antihypertensive, to her not having a disabling stroke at 72. She was treated for early-stage breast cancer at 68 and remains in remission. She also takes a proton pump inhibitor, an SSRI begun after her husband's death 3 years ago, thyroid replacement, a bisphosphonate, and a sleep aid. She has mild cognitive impairment that her daughter has noticed, but she has not. She has substantial untreated hearing loss, and she has not exercised regularly in 20 years. She lives alone.

Nothing about her is anomalous. She is approximately what the program promised: long life past the diseases it learned to treat. What that program offers her now is a target-by-target approach whose aggregate is a polypharmacy regimen with imperfectly characterized interactions and a net benefit, in those over 80, not established by trials that largely excluded patients like her (105, 106). Her cognitive impairment is, on the dominant framing, a candidate for anti-amyloid agents of modest effect and non-trivial adverse-event profile (19, 20); her depression, a candidate for further pharmacological adjustment on mixed evidence (107). Her isolation, hearing loss, sedentary behavior, and bereavement are not diseases at all on this framing, but contextual factors that may aggravate the diseases proper.

The multidomain framing counts them differently. The Lancet Commission on Dementia identifies fourteen modifiable risk factors that, addressed across the life course, may account for approximately 45% of dementia cases worldwide (108)—among them hearing loss, depression, physical inactivity, social isolation, untreated vision loss, and elevated LDL cholesterol—and this patient carries several. The FINGER family of trials has shown that coordinated interventions across diet, physical activity, cognitive engagement, and vascular risk can slow cognitive decline in at-risk older adults, though modestly and with demanding implementation requirements (42–44). The infrastructure for delivering that coordination—hearing aids, exercise programs, sleep and nutritional support, vision care, social engagement—is fragmented, poorly reimbursed, and unevenly available, while billions have gone to agents whose effect on her trajectory will, at best, be marginal. She receives what the architecture provides. The architecture was not built for her.

## The neuropathy nobody can localize

If the success–failure inversion has a clinical face more legible than dementia, it is the late-life patient with progressive numbness in the feet, unsteadiness in the dark, and a workup that terminates in the word *idiopathic*. Peripheral neuropathy is among the most prevalent neurological conditions of later life, affecting an estimated 7% of the general population and rising to between a quarter and a half of adults over 65, depending on case ascertainment and the threshold for clinical vs. subclinical involvement (55, 56). It is a leading contributor to falls, gait disturbance, neuropathic pain, and loss of independence in older adults, and its public health burden is comparable in magnitude to the more prominent neurodegenerative conditions, though it receives a small fraction of the research attention (57, 58).

The diagnostic workup for distal symmetric polyneuropathy is the localization program operating in textbook form. The clinician identifies the syndrome, characterizes its distribution and electrophysiology, and screens for the standard list of identifiable causes: diabetes, prediabetes, vitamin B12 deficiency, alcohol exposure, paraproteinemia, thyroid disease, renal failure, HIV, autoimmune neuropathies, hereditary neuropathies, and toxic or iatrogenic exposures, including chemotherapy. The algorithm works well when one of these causes is present, and treatment of the identified cause—glycemic control, B12 repletion, alcohol cessation, immunomodulation in the autoimmune variants—can arrest or partially reverse the process. A meaningful fraction of late-life neuropathy has accordingly become treatable, and this is a genuine, if partial, success.

What the algorithm cannot do is account for the substantial fraction of cases in which no specific cause is identified after a thorough evaluation. Estimates vary by series and by the rigor of the workup, but between 25 and 50% of distal symmetric polyneuropathy in older adults is classified as idiopathic or cryptogenic (59, 60). The terminology is honest: the clinician has run the localization algorithm to completion, and it has not localized. The patient still has measurable nerve conduction abnormalities, characteristic small-fiber loss on skin biopsy when performed, real symptoms, and a trajectory of progression. The disease is present; the address is not.

The candidate mechanisms read like a catalog of the hallmarks of aging operating on the peripheral nerve: axonal dying-back reflecting the bioenergetic vulnerability of the body's longest neurons, whose maintenance depends on mitochondrial transport over distances approaching a meter (61, 62); Schwann cell senescence and impaired remyelination (63, 64); microvascular rarefaction of the vasa nervorum (65); advanced glycation end-products stiffening the extracellular matrix even without frank hyperglycemia (66); subclinical metabolic syndrome at glucose levels below the diabetic threshold (67, 68); and low-grade inflammation, oxidative stress, and possibly gut-derived metabolites, none of which has emerged as primary (69). The architecture is exactly what this paper has described as resistant to localization: distributed across scales, multifactorial, decades-long, with no node carrying the necessary and sufficient relation that targeting requires.

The clinical response apparatus reflects the architectural mismatch. The patient with idiopathic neuropathy is typically offered symptomatic pharmacotherapy—gabapentinoids, duloxetine, tricyclic antidepressants, topical agents—whose number-needed-to-treat for meaningful pain reduction is four to seven, whose tolerability in older adults is poor, and whose effects on the underlying neuropathic process are nil (70, 71). The interventions with the strongest evidence for affecting disease trajectory rather than symptoms—aerobic and resistance exercise, weight loss in the metabolically vulnerable, glycemic optimization at pre-diabetic levels, correction of subclinical nutritional deficiencies, and fall-prevention programs addressing the functional consequences—are multimodal, behavioral, unpatentable, poorly reimbursed, and structurally disfavored by the apparatus producing and evaluating therapeutic interventions (72–74). The pharmaceutical pipeline for disease-modifying neuropathy treatment has been, with few exceptions, a sequence of failed trials of agents targeting individual mechanisms in isolation (75).

Generalized late-life weakness and sarcopenia exhibit the same pattern in a different tissue. Loss of muscle mass and function is among the most consistent predictors of disability, falls, hospitalization, and mortality in older adults, and its biology spans every hallmark of aging (76, 77). Two decades of pharmacological development—myostatin inhibitors, selective androgen receptor modulators, ghrelin agonists, repurposed ACE inhibitors—have produced no agent that meaningfully alters the trajectory in clinical use (78, 79), while the interventions with the largest measured effects are progressive resistance training and adequate dietary protein, with vitamin D repletion in the deficient (45–47). These are inexpensive, scalable in principle, and structurally incompatible with the apparatus that funds drug development, runs regulatory trials, and reimburses care. The intervention that works is the one the system is least equipped to deliver.

These conditions interact, and the interaction sharpens the institutional point. The composite patient of Box 1 almost certainly carries some polyneuropathy and sarcopenia alongside her cognitive impairment: the neuropathy worsens her balance, the sarcopenia reduces her capacity to recover from perturbation, the cognitive impairment limits her engagement with the exercise and nutritional interventions that would help, and the polypharmacy contributes to all three. She is told, in effect, that there is nothing specifically wrong and nothing specifically to do—the clinical translation of a localization apparatus that has run its algorithm and found no target. The apparatus offers her a prescription instead, because that is what it is built to provide.

Idiopathic neuropathy and generalized late-life weakness are not exotic edge cases. They are among the most common and disabling reasons older adults lose independence, and they exemplify with unusual clarity what the paper has called the residual category: real diseases, with measurable pathology, that the localization program has examined and been unable to address, and for which the institutional apparatus continues to produce iterations of the only kind of intervention it knows how to produce. The patient is told her neuropathy is idiopathic, and her weakness is age-related. Both statements are clinically true and analytically incomplete. They describe the program's limit rather than the disease's.

## What it would take to fund a disease without an address

Suppose an institutional architect were tasked with designing a research funding apparatus appropriate to the diseases the existing apparatus has filtered into the surviving population. Six requirements follow from the structural mismatches described above, and the objection that they amount to a wish rather than a plan is answerable. Table 4 pairs each with a specific mechanism and an existing institutional analog, on the principle that nothing here requires an instrument that has never been built—only that instruments already used elsewhere be applied to this class of problem and at commensurate scale.

**Table 4:** Required features, candidate mechanisms, and existing analogs.

Requirement	Mechanism	Existing analog
Unit of funding is the characterization, not the hypothesis	Cohort- and platform-based awards with hypotheses generated internally and reviewed on data quality and access	NIH Common Fund programs; NIA Nathan Shock Centers; UK Biobank; *All of Us*
Integrative output counts as a deliverable	Review criteria scoring multiscale characterization and data infrastructure independently of new-target discovery	Resource and infrastructure award criteria in genomics and imaging consortia
Multimodal intervention is trialable	A regulatory pathway for combined interventions with composite functional endpoints and pre-specified interaction contrasts	Factorial designs in cardiovascular prevention; the methodological groundwork of the FINGER trials (42–44)
Non-commercial interventions have a sponsor	Public sponsorship of unpatentable combination trials as a standing budget line rather than a competitive exception	Publicly funded comparative-effectiveness and pragmatic trial programs
Investigators are competent across scales	Dual-scale fellowships with joint appointment and promotion criteria written for breadth	Physician–scientist training structures; interdisciplinary center appointments
Funding horizon matches process horizon	Renewable multi-decade commitments reviewed on continuity rather than periodic novelty	Long-term observatories in astronomy and climate science; the historical funding of Framingham and the Rotterdam Study

Each row lists a structural requirement for research on multiscale, temporally extended processes, a mechanism meeting it, and an existing instrument demonstrating institutional feasibility. The claim is not that these analogs serve this purpose, but that they establish no design obstacle

The political economy should be stated plainly, since it determines which of these is achievable and in what order. Disease-designated appropriation has an organized constituency: patient advocacy organizations, disease-specific professional societies, and legislators with a nameable achievement to claim. Process-directed funding has almost none, because the beneficiary population is everyone eventually and no one currently, which is the classic structure of a diffuse interest facing concentrated ones. This asymmetry, rather than any scientific disagreement, is the most likely explanation for why repeated recommendations for realignment have not been acted on, and it implies that the realistic sequence begins with the mechanisms that require no new appropriation and no reallocation away from existing constituencies—review criteria, training structures, trial methodology, and regulatory pathway—rather than with the budgetary reallocation that would require defeating them.

A recent whole-body characterization illustrates what the alternative apparatus can produce. (80) performed unbiased segmentation of peripheral nerves, immune cells, and 31 organs and tissues across the entire mouse body at cell-level resolution. By refusing to specify in advance which nerves to examine, the analysis identified an obesity-associated trigeminal alteration that no hypothesis-driven study could have generated. Building institutional support for such work at scale—imaging infrastructure, data infrastructure, computational expertise, multi-investigator integration, and evaluation criteria that reward characterization rather than mechanism alone—is a concrete instance of what reorienting the funding apparatus would require.

None of these features is novel; all have been proposed for decades. The question is why they have not taken hold at a scale commensurate with the disease burden. Three mechanisms are identifiable. Longitudinal cohort infrastructure requires commitment across funding cycles and administrations that the project-based mechanism does not naturally support and that competing near-term priorities repeatedly erode. The dependence is not hypothetical: analyses of the consequences of withdrawing United States public funding indicate how much of the global research effort rests on a small number of sustained public commitments, and how quickly capacity contracts when they are interrupted (48). Multimodal trial methodology has developed slowly because no commercial sponsor is interested in demonstrating that a combination of unpatentable interventions outperforms any single product in isolation. Cross-scale training is constrained by an academic labor market that rewards depth within a specialty and promotion criteria that penalize breadth. These are structural features of mutually reinforcing institutions: funding mechanisms, regulatory pathways, training structures, and evaluation criteria, each stabilizing the others so that marginal adjustments in one are absorbed by the rest. The proposal is not that the institutions should try harder within their current architecture; rather, the architecture itself requires simultaneous reorganization across its components. The localization framework produced the apparatus that now reproduces it. The diseases that escape the program will not be addressed by adjustments within it; they require a different apparatus, with different metrics, different timescales, and different relationships among the institutions involved. Whether such an apparatus can be built within the political economy of contemporary biomedical research is an open question.

## The diagnostic precondition

The institutional argument so far has emphasized the therapeutic asymmetry: the program produces interventions for diseases it can localize and struggles to produce interventions for diseases it cannot. There is a parallel asymmetry upstream of intervention, in what the program can *see*, and the diagnostic limitation may be the more consequential. Clinicians are, with good reason, reluctant to treat patients without visible pathology. The trial apparatus is reluctant to evaluate interventions without a measurable disease. The reimbursement apparatus is structurally incapable of paying for interventions that prevent conditions before those conditions become coded diagnoses. The cumulative effect is that diseases are addressed at the point of irreversible tissue damage, even when the underlying processes have been developing for decades, because the apparatus has no category for the decades in which they were developing invisibly. The diseases of long life begin at the level of single cells, and the apparatus can act only at the level of organ failure.

The asymmetry is clearest in conditions for which late-stage interventions are themselves expensive, only partially effective, and routinely reimbursed because the alternative is catastrophic. Coronary stenting after myocardial infarction restores patency to an artery that should not have closed, decades after the lipid deposition that closed it began. Surgical resection and adjuvant chemotherapy address tumors that became clinically detectable years to decades after the founding mutational events. Anti-vascular endothelial growth factor agents arrest the neovascularization that defines wet age-related macular degeneration and are reimbursed accordingly (81–83), because a patient who can no longer see imposes costs far exceeding those of repeated intravitreal injections. Each intervention is genuine, and the reimbursement logic is sound within the framework in which it operates. What the framework leaves outside reimbursement, institutional delivery, and routine clinical workflow is the decades-earlier intervention that could prevent the patient from reaching the point at which late-stage intervention becomes necessary. Box 2 develops macular degeneration as a case in which the enabling diagnostic technology is already maturing, while the institutional architecture to deploy it is absent.

Box 2 **Seeing before the disease presents: the retina as a case.**

Polarization-sensitive optical coherence tomography and related techniques for non-invasive imaging of the living retina at cellular resolution suggest that the earliest structural fingerprints of macular degeneration may be detectable four to five decades before clinical disease, before the appearance of drusen, and at a point at which the retina appears healthy by conventional examination (109). At that point, low-cost interventions with reasonable evidence—photobiomodulation, the Mediterranean dietary pattern, and the antioxidant and zinc regimens studied in AREDS2—may plausibly slow progression by years or decades (110–112). The patient who receives them in the fifth decade of life may avoid injections in the 8th. The technology to identify that population exists in research settings; the institutional architecture to deploy it at a population scale, to reimburse the early intervention, and to evaluate its long-term consequences does not.

The retina is a privileged site for such work, and the reasons generalize. The tissue holds neural elements, vasculature, connective tissue, and immune-related processes in close apposition while remaining optically accessible *in vivo* through the pupil. Adaptive optics combined with ultra-high-resolution polarization-sensitive optical coherence tomography (113–115) permits visualization of microscopic morphology at or approaching single-cell resolution in a living human eye, without biopsy or contrast. Beyond static morphology, such systems can quantify dynamic tissue behavior—how the cardiac pulse transiently alters birefringence, fast-axis orientation, or tissue deformation—yielding candidate biomarkers for fibrosis, calcification, vascular stiffness, and tissue aging long before overt disease develops (116, 117). One modality applied to a single accessible tissue can, in principle, deliver readouts across several hallmarks of aging and several organ systems whose disease processes the localization framework studies separately, one at a time.

Longitudinal imaging is the natural extension. Following patients over decades at cellular resolution would document gradual cell loss, tissue remodeling, and microvascular rarefaction beyond the resolution of conventional clinical methods, with deep learning as the necessary infrastructure because the relevant signals are subtle, distributed across the image, and unlikely to be specifiable in advance. Combined with the multi-decade cohort infrastructure described above, the resulting datasets could generate individualized risk estimates for dementia, coronary artery disease, stroke, Parkinson's disease, and Alzheimer's disease. The predictions would be derived from microscopic retinal fingerprints learned across prior populations. The proposal is unrealized at scale, and its technical, methodological, and ethical preconditions remain considerable. Structurally, however, it is of a kind with the whole-body characterization (80) demonstrated in mice: unbiased, multiscale, hypothesis-generating, dependent on computational integration of large image datasets, and incompatible with funding mechanisms that require a single mechanism to be specified in advance.

The role of machine learning in this program deserves a more precise statement than it usually receives, because it cuts both ways. Methods that discover structure without pre-specified features are well matched to phenomena that resist prior localization: they can operate on the full measurement space rather than on a hypothesis about which part of it matters, which is exactly the constraint that hypothesis-driven design imposes and that multiscale phenomena violate. But the same apparatus this paper describes will tend to absorb these methods as target-discovery engines, since a model that surfaces a novel, specifiable, perturbable candidate mechanism produces exactly the output the evaluative criteria reward. A system trained to find the most tractable target reproduces second-order fragmentation at higher throughput, and does so with the appearance of unbiased discovery. Whether these methods accelerate integration or decomposition, therefore, depends not on the methods but on what their outputs are evaluated against—which is to say that they offer no escape from the problem this paper describes, and are subject to it.

The conceptual shift this work would require can be stated plainly. Twentieth-century medicine learned to react to clinically visible disease. The medicine appropriate to the longest-lived population in human history will have to learn to monitor microscopic tissue health continuously, across decades, before the irreversible pathology develops that the current apparatus is built to address. The diagnostic precondition for the multiscale medicine described in this paper is the capacity to see multiscale disease before it presents as organ failure. The diseases that resist localization may not resist visualization, given the right instruments and the institutional commitment to deploy them before symptoms appear.

## Geroscience and the hallmarks: integration or a longer list of targets?

The geroscience framework is the most developed available response to first-order fragmentation, and the argument of this paper therefore turns on whether it can withstand the second. By proposing that the major chronic diseases of late life share upstream biology in the form of the hallmarks of aging, the framework unifies conditions that current nosology treats separately, and by targeting that biology rather than the diseases, it offers an intervention strategy that does not require disease-by-disease development (40, 41).

### Why the hallmarks and not the alternatives

The hallmarks framework is not the only model of aging on offer, and the alternatives partition the explanatory space differently, with different implications for what research and funding should look like. Damage-based approaches, including the SENS program, treat aging as accumulated damage and license a repair-oriented strategy organized by damage class rather than by process (84, 85). Hyperfunction and quasi-programmed accounts treat late-life pathology as the continued operation of developmental programs rather than as damage, thereby relocating the intervention target entirely (86, 87). Network models, resilience-centered frameworks, and accounts organized around the loss of homeostatic integration make the relevant object a system-level property that no list of components can capture (88, 89). Inflammaging posits a single, unifying process rather than a list (90). We do not adjudicate among these, and our focus on the hallmarks is not a claim that they are correct or best supported. It follows instead from the object of this paper. Our argument concerns the interaction between conceptual frameworks and institutional architectures, and the hallmarks framework is the formulation that has actually acquired institutional traction: it organizes funding announcements, consortium structures, trial rationales, review panels, and training programs in a way the alternatives do not. For an argument about what institutions do to frameworks, uptake is the relevant selection criterion.

### What the framework is

The critical literature has pressed a set of questions to which the framework's institutional function is sensitive, and we take them in turn. First, are the hallmarks causes, mechanisms, manifestations, risk factors, or heuristic categories? They are heterogeneous in kind, and the framework does not itself distinguish them. (91) argues that the list mixes drivers with passengers, and the distinction has direct funding consequences: a passenger mechanism makes an attractive target—measurable, perturbable, responsive—and a poor one, and nothing in the framework's criteria for hallmark status separates the two. Second, are they causally independent, hierarchically organized, or mutually constitutive? The framework's own presentation describes extensive interconnections, but it specifies no causal topology, so it cannot say when an intervention at one element is expected to propagate to others (92). Third, does the list constitute a unified research program or a shared vocabulary? On the criteria that distinguish these—does it generate novel predictions, does it specify what would disconfirm hallmark status, does it constrain the space of admissible interventions—the framework at present functions closer to a shared vocabulary, and (93) develops this assessment in detail.

### What the framework does institutionally

Fourth, can interventions directed at individual hallmarks escape the problems attributed to disease-based reductionism? On the analysis of this paper, they cannot, because they instantiate second-order fragmentation directly: a single-hallmark intervention has a single mechanism of action, a bounded time horizon, a patentable composition, and a pre-specifiable endpoint, which is to say it satisfies every criterion that produced first-order fragmentation in the first place. Fifth, does expansion of the list favor integration? The extension from nine hallmarks to twelve added candidate targets more clearly than it added integrative constraint, and each addition is independently fundable in a way that their conjunction is not. Sixth—and this is the question on which the framework's future turns—what would count as evidence that combined intervention on several hallmarks is genuinely superior to intervention on one? The answer is reasonably clear: factorial or otherwise interaction-powered designs, shared endpoints across arms, and pre-specified criteria for superadditivity rather than *posthoc* comparison of separately conducted trials. Such designs are, to our knowledge, nearly absent from the registered literature, and that absence is not evidence against the combinatorial hypothesis (94); it is evidence that the hypothesis has not been tested, and the reason it has not been tested is the subject of this paper. (95) document the specific obstacles—the absence of an approvable aging endpoint, the composite-outcome problem, the duration mismatch, the sponsor problem for unpatentable combinations—and (96) trace the resulting bottleneck between geroscience and healthcare delivery.

What geroscience has accomplished, with value, is to legitimize the framing of late-life disease as a multiscale phenomenon within the biomedical mainstream, and the progress is not rhetorical: infrastructure, international consortia, aging-related trial methodology (97), and a generation of investigators trained to think across hallmarks rather than within one. Whether the framework becomes an integrative program or a longer list of targets is not, on the analysis offered here, a question about the framework. It is a question about whether the apparatus that funds it can evaluate a claim about interaction.

## Longevity, morbidity, and what follows from neither

The argument to this point has been institutional, and its extension to the experience of long life requires care, because several claims in this vicinity are routinely run together and do not entail one another. We therefore state what we assert, what we treat as conditional, and what we decline.

We assert three descriptive claims. Aging increases vulnerability to disease. Longer lives increase cumulative exposure to age-related pathology. Life extension may, depending on what is extended, include lengthened periods of morbidity, and the empirical question of whether observed gains in life expectancy have been accompanied by proportional gains in healthy life expectancy remains genuinely contested. We treat as conditional the claim that prolonging life without prolonging health produces ethically problematic outcomes: this is precisely what our argument makes institution-dependent, since the morbidity in question is substantially a function of whether the apparatus can deliver the multidomain interventions that the architecture of these conditions calls for.

We decline three further claims. We take no position on whether aging is biologically harmful in the evaluative sense, on whether aging is undesirable, or on whether society is under an obligation to intervene in aging. These are substantive questions with a serious and unresolved literature (98–102), our institutional argument does not turn on them, and a paper that smuggled answers to them through its tone rather than its arguments would be doing something we would criticize elsewhere. The claim that biomedical institutions have not adapted to the forms of vulnerability accompanying longer lives is compatible with every position in that debate, which is one of its merits.

With those distinctions fixed, people now routinely live past 85, past 90, past 100, often with intact cognition through much of that span. What the 20th century implicitly promised was that long life would resemble middle life, only longer. What frequently arrives instead is the slow disclosure of a multiscale, distributed, contextually sustained pathology in a body that remains otherwise functional—and, critically, a healthcare system organized around discrete diagnoses, discrete medications, and discrete encounters, which is the clinical face of the same fragmentation this paper has traced through the funding apparatus.

Each specialty addresses one organ, and no specialty addresses the patient. The medications listed in Box 1 were developed to address specific lesions, each plausibly necessary at the time of prescription, none designed for the seventh or eighth simultaneous prescription, none evaluated in trials that included people like her. The recognition that her condition is multiscale does not, by itself, change the prescription, the appointment structure, or the reimbursement code. The recognition is acknowledged in geriatric texts and silently over-ridden in everyday geriatric practice.

The cultural framing is similarly unprepared. Twentieth-century medicine framed disease as an enemy to be defeated and longevity as the prize of victory, which made sense when the diseases were discrete enemies amenable to defeat. It makes less sense when the conditions of long life are not enemies but consequences—distributed states the multiscale system enters as it ages, with feedback among components and interactions with environments not designed for it. The 84-year-old with mild cognitive impairment, hearing loss, isolation, polypharmacy, and grief faces no enemy; she inhabits a state. Conquest has no easy translation into accompaniment, and the alternative framing—articulated for decades within geriatrics and palliative care, treating these conditions as features of an aged biological system and aiming intervention at function, capacity, and quality of life in interaction with the patient's own values and circumstances (103, 104)—carries no claim to novelty. What is new is the convergence of its correctness with the demographic situation that makes it urgent, and it has yet to become the dominant organizing principle of either medicine or research.

One qualification is essential here, and we want to state it in stronger terms than the framing of “conquest vs. accompaniment” might suggest. Accompaniment is not a substitute for prevention or for biological intervention, and nothing in this paper should be read as recommending care in place of repair. A pluralist medicine of aging would combine prevention, biological intervention, repair, care, adaptation, and social support, and these stand in complementary rather than competing relations. Our contrast with the conquest framing concerns the *sufficiency* of the single-target model, not the legitimacy of biological intervention as such. The divide-and-conquer strategy for age-related functional decline is a serious position that our argument must engage rather than assume away (84), and engaging it sharpens the claim: the objection is not that aging should not be decomposed into tractable sub-problems, but that the institutional implementation of decomposition has produced an evaluative regime with no procedure for recomposing what it decomposes, and no mechanism for funding the test of whether recomposition helps.

A disease that resists localization is not a research failure. It is what a distributed causal architecture looks like from the clinic, and medicine's obligation to engage with it does not diminish simply because engagement is harder to fund, trial, or reimburse.

## A final inversion

We have argued that the diseases medicine has solved share an architecture, that the diseases it has not solved share a different one, and that the institutional apparatus which produced the solutions is structurally unsuited to producing solutions for the residual. Four claims carry the argument.

First, the sorting of diseases by tractability was structural rather than contingent, and it is the premise of the argument rather than its conclusion. What follows from it is not a prediction about the next breakthrough but a question about institutions: whether an apparatus calibrated to the first architecture can fund, evaluate, and reward research organized around the second.

Second, that apparatus is self-reproducing and currently faces no rival with comparable institutional purchase. Grants, journals, trials, careers, and clinical training each select for compatibility with the localization program, so investigators who would otherwise work differently adapt, and the adaptations preserve the program even where the science does not support it. The biopsychosocial model, network theories of psychopathology, geroscience, multimorbidity initiatives, and multidomain prevention trials each contribute to an emerging alternative, but none has yet matched the incumbent's conceptual clarity, methodological tractability, regulatory acceptability, and clinical actionability at once—which is what it takes to displace a degenerating program.

Third, the resources needed to build that alternative are organizational, methodological, regulatory, pedagogical, and cultural before they are monetary. Even a doubled NIH budget would be absorbed by the existing apparatus within its familiar categories. The required change lies in the categories themselves—in what counts as disease, what counts as evidence, what counts as a successful research program, what counts as a reimbursable intervention, and at what point in a multi-decade trajectory the medical apparatus is permitted to act. The diagnostic precondition examined above is one concrete instance; the funding mechanisms of Table 4 are another.

Fourth, the experience of being old in the longest-lived population in human history is shaped by the same architecture that shapes the research. The fragmentation that frustrates the patient of Box 1 and her clinicians reflects how the field categorizes its subject matter rather than a failure of implementation, and the reframing articulated by geriatrics and palliative care will not become dominant without parallel change in the institutions that organize medical knowledge and care.

These claims prescribe no course of action. What they license is an inversion of the standard framing. On the standard story, the diseases medicine has addressed are paradigm cases of what medicine can do in principle, and the diseases not yet addressed are cases it will address in due course, once the science has progressed sufficiently: the successes set the expectation, and the failures are continuations of the same story awaiting their breakthrough. We have argued the reverse. The successes were exhausting a finite class of structurally tractable problems, and the failures were not waypoints toward future success but indications of a class of problems the program cannot address. The standard story is accurate in its description of the successes and mistaken in its extrapolation. The next phase of medicine, if it is to address the diseases of a long-lived population, will not look like an extension of the program that produced them. It will have to retain targeted intervention where the architecture supports it and add what the architecture requires elsewhere—multiscale characterization, combinatorial rather than single-target perturbation, sustained measurement over decades, and contextual care. The addition is what remains unbuilt; it is not a replacement for what was built.

The patients are here, in numbers no previous era of medicine has seen, and that they are here is the achievement rather than the problem. Their conditions have an architecture that the dominant evaluative criteria cannot register: no single address, no bounded time horizon, and no mechanism of action that can be stated in the form a review panel is trained to recognize. The question is not whether those conditions can be localized. It is whether the institutions that fund, evaluate, and reward biomedical research can be redesigned for the disease landscape they have produced rather than the one they were built to address. The answer will be visible only in retrospect: in which research programs were funded and which were not, which investigators were promoted and which were passed over, and which clinical encounters were reimbursed and which were left to the unpaid labor of families and overburdened caregivers.

We hope they can. We are not certain they will
